# Editorial: Evolution & Genomic Adaptation of Emerging and Re-emerging RNA Viruses

**DOI:** 10.3389/fmicb.2021.777257

**Published:** 2021-11-12

**Authors:** Kai Huang, Justin Jang Hann Chu

**Affiliations:** ^1^Department of Pathology, University of Texas Medical Branch, Galveston, TX, United States; ^2^Galveston National Laboratory, University of Texas Medical Branch, Galveston, TX, United States; ^3^Laboratory of Molecular RNA Virology and Antiviral Strategies, Department of Microbiology and Immunology, Yong Loo Lin School of Medicine, National University of Singapore, Singapore, Singapore; ^4^Infectious Diseases Translational Research Program, Yong Loo Lin School of Medicine, National University of Singapore, Singapore, Singapore; ^5^Collaborative and Translation Unit for HFMD, Institute of Molecular and Cell Biology, Agency for Science, Technology and Research, Singapore, Singapore

**Keywords:** RNA virus, molecular epidemiology, phylogeny, evolution, emerging/re-emerging infections

Emerging and re-emerging infectious diseases are defined as diseases caused by unidentified and reappearing pathogens (NIAID, 2018). RNA viruses are mostly responsible for most of such infectious disease outbreaks (Nichol et al., 2000). One of the key reasons is due to genomic alterations such as spontaneous mutation recombination or reassortment which occurs during adaptation and evolution processes (Nichol et al., 2000). When those genomic changes have been accumulated to a certain level or when the changes are on the antigenic or receptor-binding region, the host immune systems are no longer able to recognize the new variants, resulting in global viral outbreaks (De Wit et al., 2016; Nelemans and Kikkert, 2019; Kikkert, 2020). SARS-CoV-2 and its variants has claimed more than 4.56 million lives from December 2019 until the writing of this manuscript. The high mortality rate emphasizes the importance of continuously monitoring these emerging viruses' evolution and genomic adaptation (WHO, 2021).

The purpose of this Research Topic serves to provide an open access platform for an international global team of multidisciplinary researchers and scientists to share their findings on the viral genomic feathers and changes, to provide a platform enabling public health officials to warn the global community of potential and existing epidemics and pandemics, and to develop an analytical platform for researchers to evaluate the outbreak risks, to prepare and to control future pandemics (Huang et al., 2021). In this Research Topic, the authors will present their most recent genomic investigations on these viruses. A total of 28 manuscripts including original research and review have been received, of which 15 were eventually accepted for journal publications after rigorous peer review processes. Based on the viruses involved, these 15 articles can be briefly classified into three groups: positive sense single-strand RNA (+ssRNA) viruses; negative-sense single-strand RNA (-ssRNA) viruses; diagnosis and dynamic single-strand RNA Virus-Host interactions.

The first group +ssRNA viruses are the Group IV viruses in the Baltimore classification system (Baltimore, 1971; Cann, 2016), including viruses from *Coronaviridae, Picornaviridae, Flaviviridae, Togaviridae*. Within this group viruses, Coronavirus is the most researched virus in this topic. Three original research articles discussed the SARS-CoV-2 coronaviruses identified in China, Brazil, and Uruguay, respectively. In China, Song et al. isolated SARS-CoV-2 viruses from the Henan Province, which is adjacent to the Hubei Province, the region which has the highest mortality rate due to the SARS-CoV-2 pandemic within China. They analyzed the samples from different locations to estimate the virus's most recent common ancestor (TMRCA) and evolutionary rate. In Brazil, Resende et al. analyzed 190 SARS-CoV-2 viruses isolated from 13 Brazilian states and found the B.1.1.33-like viruses circulating in Brazil might have been transmitted from Europe or domestically erupted a few weeks before regional outbreaks. Their analysis also indicates public health interventions were successful because the median effective reproductive number (Re) dropped by 66%. In Uruguay, Mir et al. investigated the local virus source and the transmission rate(s). Based on the 122 viruses recovered at Brazilian–Uruguayan border area, they found that the SARS-CoV-2 viruses in the Uruguay border were introduced multiple times independently from Brazil (lineage B.1.1.28 and B.1.1.33). The researchers also revealed in their research that the synonymous and non-synonymous single nucleotide polymorphisms (SNP) are the genetic variations responsible to define the lineages. Castonguay et al. completed the fourth SARS-CoV-2 meta-analysis, which systematically tracked the evolutionary trajectory of SARS-CoV-2 over time, identified emerging mutations, and modeled the structural changes and corresponding molecular interactions. The fifth coronavirus is the avian infectious bronchitis virus (IBV). Jiang et al. identified a critical mutation to determine the host tropism alteration, which is a valuable key in understanding why the coronavirus could jump from one host to another. Porcine Epidemic Diarrhea virus (PEDV) is the last coronavirus discussed in this Research Topic. Li et al. isolated a PEDV, which has been detected to contain a unique insertion in the S1 protein binding by the recombination test. They predicted the structure of this new recombinant and proved its biological correlation by showing this strain had higher pathogenicity than the other viruses isolated in the piglet *in vivo* challenge.

Following the *Coronaviridae, Picornaviridae* is the second most popular virus family within this topic. In two articles, researchers investigated the Norovirus and Coxsackievirus, which are enteroviruses within the family *Picornaviridae*. Zuo et al. identified the new Norovirus GII.17 variants, which surpassed the predominant GII.4 genotype causing the Kawasaki variant outbreaks in 2014–2015. Serological analysis showed weak cross-protection to these new variants so attention should be taken to prevent future outbreaks. The corresponding mutated amino acids on antigenic sites were also identified. The second study led by Li's team focused on the Norovirus GII.2 clusters, which caused unprecedented endemic outbreaks in 2016–2017. Eight distinct clusters with increased genetic diversity were characterized with an absence of elevated evolutionary rate. Additionally, the selection pressure was detected, suggesting the outbreak was probably not related to an evolutionary adaptation. The second virus in the *Picornaviridae*, is the Coxsackievirus A16 (CVA16) and was reported by Nhu et al. This molecular epidemiology of CVA16 in Southern Vietnam was documented for the first time. They found the Vietnamese CVA16 strains belong to a single genogroup B1a and these viruses displayed a less pronounced genetic alternation compared to the enterovirus A71 (EV-A71). Zika virus (ZIKV) of the *Flaviviridae* family and Chikungunya virus (CHIKV) of the *Togaviridae* family are the last two members of ssRNA viruses mentioned in this article collection. Su-Jhen Hung and Sheng-Wen Huang reviewed the amino acid substitutions and factors contributing to the pathogenicity and transmission of ZIKV. Sharif et al. systematically reviewed the evolution, epidemiology, phylogeny for CHIKV.

Influenza virus is the major representative of negative-sense ssRNA viruses, the Group V Baltimore viruses outlined in this topic. Influenza had caused at least 5 pandemics since the 20th century (Kilbourne, 2006). Reassortments of segmented genes from different sources were generally believed to be responsible for these prior pandemics. Trifkovic et al. investigated the gene reassortment driving force using the vaccine seed production model. They found a selective preference for genome compatibility between different viruses. They also found a powerful driving force to determine the dominant progeny besides the ones they initially thought. In the second influenza paper, Guo et al. characterized the interspecies transmission process from the original avian host to a new canine host. They identified 54 substituted amino acids fixed during the interspecies transmission. By analyzing the selection pressure and codon usage, they found the canine influenza viruses are better adapted to avian hosts, supporting the theory of their avian origin.

In the third Research Topic group, the virus diagnosis and host-virus interactive network were discussed. Based on the conservative RNA gene segment, Han et al. developed an easy and fast isothermal amplification assay to detect RVFV with high sensitivity and specificity. The test uses a strip that can detect RVFV at as low as ~200 copies/μL, 100-fold, which is more sensitive than real-time RT-PCR assay without cross-reactivity to viruses causing similar symptoms. The test requires no specific equipment and can be done in 1 h with results visible in 5 min. Such a quick test with high sensitivity and specificity is quite prominent in the detection of emerging pathogens. In the second paper in the group, Romano et al. proposed a multidisciplinary framework using Systems Thinking (ST) to study the dynamics of host-virus interactive networks for the first time. In the Systems Thinking theory, changes of one component in the network will lead to the corresponding changes of other components until it reaches a new balanced stationary status. Using the System Dynamics (SD) modeling, the authors computationally simulated the dynamic trajectory of host-virus interactions and identified the leverage points to minimize virion release.

In summary, this Research Topic provides cutting edge methodologies, research, and practices in the *Evolution & Genomic Adaptation of Emerging and Re-emerging RNA Viruses*. We heartily acknowledge and are grateful for the contributions and outstanding works of all the authors and reviewers of this Research Topic. We believe this collection will raise the awareness of emerging/re-emerging RNA viruses and enable proficient monitoring processes in order to decrease mortality rates by having an efficient system in place to control the next SARS-CoV-2 outbreak.

## Author Contributions

All authors contributed to co-editing the special Research Topic, edited, revised, and approved the editorial.

## Funding

This work was supported by the Centers for Excellence in Influenza Research and Surveillance (CEIRS) Program, Grant Number HHSN272201400008C, from the National Institute of Allergy and Infectious Diseases (NIAID).

## Conflict of Interest

The authors declare that the research was conducted in the absence of any commercial or financial relationships that could be construed as a potential conflict of interest.

## Publisher's Note

All claims expressed in this article are solely those of the authors and do not necessarily represent those of their affiliated organizations, or those of the publisher, the editors and the reviewers. Any product that may be evaluated in this article, or claim that may be made by its manufacturer, is not guaranteed or endorsed by the publisher.
